# Exploring Different Methods for Eco‐Friendly Wool Dyeing with Natural *Carmine Dye* and Waste Wool Hydrolysates

**DOI:** 10.1002/gch2.202500165

**Published:** 2025-07-25

**Authors:** Roberta Peila, Maria Laura Tummino

**Affiliations:** ^1^ Institute of Intelligent Industrial Technologies and Systems for Advanced Manufacturing (STIIMA) National Research Council of Italy (CNR) Corso Giuseppe Pella 16 Biella 13900 Italy

**Keywords:** eco‐friendly dyeing, natural dye, waste‐wool hydrolysates, wool fabrics

## Abstract

Waste wool hydrolysates (WWHs), a by‐product originating from the alkaline hydrolysis of waste wool, are recovered and employed as auxiliaries in wool dyeing. In view of an eco‐friendly dyeing procedure, a natural dye, *Carmine*, is selected to dye wool fabrics. Different methodologies for performing the dyeing process are described. In the first procedure, the wool fabrics are pretreated with a water suspension of the WWHs at room temperature, left overnight, and then cured at 180 °C. In the second procedure, the wool fabrics are immersed in the WWH's suspension at 100 °C, dried in an oven, and subsequently dyed through the exhaustion method. In the last procedure, the WWHs are added directly to the dyeing liquor. Dye exhaustion, color coordinates, and K/S are measured to evaluate the dyeing efficiency. The dyed fabrics are also characterized in terms of thermal, chemical, mechanical and morphological properties. The results demonstrate that the WWHs are efficient alternatives to metal‐based mordants in assisting wool dyeing with *Carmine* dye. The evidence of non‐significant damages to fabrics as a consequence of the chosen treatment conditions further supports the possibility of WWH valorization in textile industries as a by‐product that otherwise would represent a waste to dispose of.

## Graphical abstract

Waste wool hydrolysates (WWHs), by‐product of the alkaline hydrolysis of waste wool are employed as coadjuvant in wool fabrics dyeing with Carmine natural dye. The results demonstrate the possibility of WWHs valorization as eco‐friendly alternative to metal‐based mordants in wool dyeing. The dyed fabrics evidence better color performances and no significant mechanical damage as a consequence of the treatment conditions.

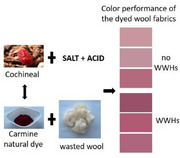

## 1 Introduction

Color is a crucial feature in human beings' lives; its importance lies in the fact that color influences our minds and emotions and the perception of the reality around us. Garment and cloth dyeing is a process employed since ancient times. It has been reported that the first primitive technologies appeared before 3000 BC.^[^ ^1^ ^]^ Initially, rudimentary techniques were developed, such as embedding colored powder into clothes or directly fixing colored plants on the fabrics. With time, these methodologies improved, and the natural pigments were extracted from the natural resources with water and then used to dye the clothes. In this way, however, only a limited number of shades were possible, and the fastness of the so‐colored garments was very poor. To favor dyeing interaction with fibers, new developments employed salt solutions of metal ions that interacted with the fibers and aided the dyeing process, also improving the dye's fastness to light and washing.^[^ ^2^, ^3^ ^]^ Many natural pigments were extracted from plants or insects. Archeological discoveries indicate examples of plants for dye extraction utilized throughout history, like *Indigofera tinctoria* and *Sambucus nigra* L. to obtain blue, Saffron, *Arbutus unedo* L. or *Cotinus coggygria* L. for yellow, *Rubia tinctorum* for red.^[^ ^4^, ^5^ ^]^ Recent literature reports different studies that employed natural dyes for textile dyeing to confer particular properties like halochromic and antibacterial.^[^ ^6^, ^7^ ^]^ Among the pigments extracted from animals, a particular interest has always been given to the Cochineal insect, which produces carminic acid, a red pigment largely employed to dye textiles. The insect originates in America and it was first used by pre‐Columbian societies.^[^ ^8^ ^]^ The intense red color of the clothes dyed with this pigment attracted the attention of the European colonists, who brought it to Europe.^[^ ^9^, ^10^ ^]^ Carminic acid's chemical structure is derived from the anthraquinone family, linked to a glucose unit. Thanks to its safety, nowadays, it has an enormous range of applications, from textiles to cosmetics and the food industry.^[^ ^11^, ^12^, ^13^ ^]^

With technological progress, however, the natural pigments employed in textile dyeing were rapidly substituted with synthesized molecules, which are less expensive and designed *ad hoc* to favor dye absorption on the different types of fibers.^[^ ^14^ ^]^ Despite these advantages, this category of dyes causes pollution and environmental concern because the excess dye not absorbed by the textile substrate is discharged into the water bodies and represents a risk not only to aquatic life but also to human health.^[^ ^15^, ^16^ ^]^ Literature reports a significant number of studies that consider dye removal from wastewater a priority for environmental safety. Adsorption, electrolysis, photocatalysis, Fenton‐based processes, ultrafiltration, and nanofiltration, plus biological methods, are the most widely explored remediation strategies.^[^ ^17^, ^18^, ^19^, ^20^, ^21^ ^]^ Due to these ecological issues, new and more eco‐friendly alternatives are under investigation. With a glance at the past, natural dyes are becoming increasingly important and are the focus of many research works. Natural molecules can be applied to different types of fibers. Protein fibers such as silk, wool, and specialty animal fibers show a higher affinity for natural dyes thanks to the aminoacidic component of their chemical structure. Naqvi et al. extracted the *Luteolin* from weld flowers to successfully dye wool and silk. To help the dyeing process, both the extracted dye and the textiles were irradiated by microwave.^[^ ^22^ ^]^ Wool and silk were dyed by Gong et al. as well with the natural pigment extracted from *Cinnamomum Camphora*.^[^ ^23^ ^]^ Mirki et al. examined the possibility of wool dyeing with *Daphne mucronata* leaf extract and different types of mordants.^[^ ^24^ ^]^ Cellulosic fibers might also be dyed with natural substances but require mordants to favor the dye absorption. Even though metal‐based mordants are often employed,^[^ ^25^, ^26^, ^27^, ^28^ ^]^ their natural counterparts are being investigated, as in the work from Pars, in which sodium alginate and gall oak were tested as bio‐mordants to dye flax and cotton fibers.^[^ ^29^ ^]^ In previous research in our laboratory, cotton and polyamide 6,6 were successfully dyed with Cochineal natural dye and Chitosan as bio‐mordant,^[^ ^30^ ^]^ changing also the cotton fabric biodegradation properties.^[^ ^31^ ^]^ Natural pigments can be applied to other synthetic fibers, as well: Fang et al. investigated the possibility of dyeing polyester at a low temperature with the natural pigment derived from bio‐waste of *Ipomoea batatas* leaves.^[^ ^32^ ^]^

In general, it is clear that the choice of dyeing process represents a key point in the textile supply chain: according to Costa et al., dyeing with natural compounds has brought about a noticeable reduction in the environmental footprint. Such impact was determined by Life Cycle Assessment (LCA), considering water, chemicals, and energy involved in the process as input parameters.^[^ ^33^ ^]^ Similarly, Eryürük et al. tinged denim fabrics with the natural dyes *Isatis tinctoria* L. and red cabbage in the presence of the natural tannic acids as mordants, finding optimized conditions for designing a sustainable process.^[^ ^34^ ^]^

Among the waste of the textile industry, wool processing by‐products and poor quality wool (that does not meet the requirements to be processed to form yarns and, in general, garments or upholstery) represent a concern.^[^ ^35^ ^]^ They are labeled as special waste of category III by the European Union,^[^ ^36^ ^]^ although they might be an important source of keratin‐based products. In many literature reports, keratin protein has indeed been extracted from waste wool for different new applications. Thanks to its structure, which is constituted of an aminoacidic backbone and disulfide links, it is able to form hydrogen bonds and electrostatic interactions with many molecules. In addition, keratin biocompatibility and bio‐functionality allow it to be used in biomedical and biotechnological fields (i.e., tissue engineering).^[^ ^37^ ^]^ Keratin hydrolysates are other biocompatible and bioactive derivatives of wool, obtained by its chemical or enzymatic hydrolysis, generally possessing a low molecular weight and an elevated number of side functional groups.^[^ ^38^ ^]^ These characteristics result from hydrolysis as an impacting process that may induce the breakage of disulfide and peptide bonds.^[^ ^39^ ^]^ In a recent paper,^[^ ^40^ ^]^ wool keratin hydrolysates formed as a by‐product of an alkaline treatment of wool (necessary to prepare adsorbent panels) were employed as dyeing coadjuvants to color cotton fabrics with natural *Carmine*. The hydrolysates were tested at neutral and acid pHs, showing that the procedure at pH 3 reached the best coloration yield, although only a pale red shade was revealed. The mechanism that rules this behavior can be summarized as follows: at acidic pH, the higher presence of available cations (NH_3_^+^) within the hydrolysate functioned as a bridge, promoting cellulose binding with the anionic *Carmine* dye. A similar effect has been described by Brouta‐Agnésa et al., who adopted hydrolyzed collagen to dye wool.^[^ ^41^ ^]^ Looking at the literature, Abo El‐Ola et al. obtained keratin hydrolysate using microwave‐assisted alkaline hydrolysis and successfully employed such as product to dye viscose fabric with Reactive Blue.^[^ ^42^ ^]^ Berechet et al. used keratin hydrolysate from sodium hydroxide alkaline hydrolysis to dye bovine leather and found an enhancement in dyeing resistance to different treatments, as well as the improvement of the specific color parameters.^[^ ^43^ ^]^

Beyond the extraction of keratin‐derived products, post‐industrial and post‐consumer wool waste can also be generated along the wool supply chain.^[^ ^44^, ^45^ ^]^ Indeed, several papers have described the impact of wool processing with tools like LCA, and have proposed several strategies to recycle wool‐based materials for new textile products.^[^ ^46^, ^47^, ^48^, ^49^, ^50^, ^51^, ^52^, ^53^, ^54^ ^]^ Waste wool could be an eco‐compatible resource in the building sector, often for insulation purposes^[^ ^55^, ^56^, ^57^, ^58^, ^59^ ^]^ or in agricultural applications (i.e., fertilizer).^[^ ^60^, ^61^ ^]^ The reasons for the research community's interest in wool valorization are, on the one hand, the polluting consequences of its landfill or incineration, with the formation of greenhouse gases.^[^ ^62^ ^]^ On the other hand, in the case of post‐industrial and post‐consumer waste, discarding wool products means dissipating all the resources needed for its processing, such as water, chemicals, energy for production and transportation and land use for animal breeding.^[^ ^63^ ^]^

Regarding the alkali reactants employed in this work to produce the hydrolysate, sodium hydroxide can raise concerns from a sustainability point of view. Eventual spills and non‐neutralized leachate derived from industrial and manufacturing activities can increase the pH of the waters that receive such alkaline residues, negatively affecting aquatic life.^[^ ^64^ ^]^ However, NaOH is commonly used in the textile sector for mercerization and dyeing/printing processes.^[^ ^65^, ^66^ ^]^ Therefore, the use of this substance for the creation of wool hydrolysate does not imply the introduction of new reagents in the production chain. Moreover, NaOH adopted in the hydrolysate recipe presented here is never discharged since it reacts with an acid to form a salt, which is, in turn, helpful in enhancing the dyeing procedure. Moreover, in our case, the employment of a strong base has been initially conceived as a method to activate the starting waste wool to become an adsorbent material.^[^ ^40^ ^]^ Only after having produced the activated wool panel, we recovered the wool hydrolysate by‐product in the water suspension, achieving an almost total zero‐waste process. It must be said that, in the absence of a preliminary procedure in which alkalis are needed, the formation of hydrolysate can be obtained by alternative strategies. Some of these are based on water‐based methods (steam explosion, superheated water) or enzymatic actions.^[^ ^67^ ^]^

Given these premises, our work is well‐integrated into the literature scenario and can give new inputs for the exploitation of waste wool derivatives. Its primary goal is to recover waste biomass derived from wool treatment to be adopted in the textile sector as an auxiliary for dyeing fabrics with natural substances and without the use of metal‐based mordants. In the literature, no evidence exists of studies evaluating the performance of wool fabrics dyed with WWHs and *Carmine* natural dye. Moreover, this work evaluates different methodologies for WWH applications, both as a pre‐treatment and directly in the dyeing liquor. The purpose is to investigate their influence on the final characteristics of the materials in terms of dyeing performance as well as physical‐chemical properties.

## 2 Experimental Section

### 2.1 Materials

**Figure 1:**
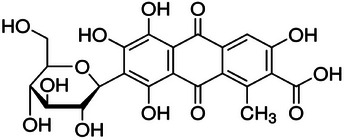
Carminic acid (natural dye).

Sodium hydroxide, NaOH 1 M solution, was purchased from Riedel‐de Haën. Acetic acid 98% and H_2_SO_4_ 95–97% were supplied by Carlo Erba. The *Carmine* natural dye (carminic acid) was kindly provided by Aromata Group Srl (Italy). Its chemical structure is reported in **Figure** **1**. The plain 100% wool fabrics, 118.87 g m^−2^_,_ were purchased from Ausiliari Tessili srl (Italy).

### 2.2 Preparation of Wool‐Derived Hydrolysates

Waste wool hydrolysis was carried out in 200 mL of NaOH 1 M water solution for 2 h at room temperature. Afterward, the solid portion was removed and the suspension containing the waste wool hydrolysates was acidified with H_2_SO_4_ till pH 3 (measured by a pH meter). The suspension was freeze‐dried and then the obtained powder was ground with a CryoMill (Retsch) at room temperature to obtain a homogeneous micropowder.

### 2.3 Wool Fabrics Functionalization with the WWHs

Three different methodologies were carried out to treat the wool fabrics with the WWHs, both as a pre‐treatment and directly into the liquor bath. In the first procedure, the WWHs were dispersed in water and the wool fabric was dipped into the suspension at room temperature and left overnight. Afterward, the fabric was manually padded till 100% wet pick up, dried in the thermostatic oven (G‐Therm 115, F.lli Galli, 1.93 kW) at 95 °C for 5 min and cured at 180 °C for 10 min. This sample will be identified as W‐1.

In the second procedure, the WWHs were dispersed in a water suspension and the wool fabric was immersed in the suspension and left at 100 °C for 1 h in a shaking water bath (Grant Scientific OLS200, ≈1 kW). The fabric was subsequently manually padded and dried in a fan oven at 30 °C. This sample will be identified as W‐2.

In the third procedure, the WWHs were dispersed into the liquor bath, without a pre‐treatment step. This sample will be identified as W‐3.

For comparison, wool fabrics were pretreated without WWHs but with 2% glacial acetic acid and/or 50 g L^−1^ Na_2_SO_4_.

### 2.4 Fabrics Dyeing

The wool fabrics treated with the WWHs were dyed using AHIBA Nuance Top Speed II equipment (Datacolor, 2.7 kW). The liquor ratio was set at 1:20 (10 g of fabric in 200 mL bath); the temperature was set at 100 °C with a heating rate of 1 °C min^−1^; the process time was 60 min. Speed was 35 rpm and reverse time 3 min. Dye concentration was 4% on the weight of fibers (owf).

Sample W‐3 was dyed by adding the wool hydrolysates directly into the liquor bath.

Other samples were prepared, namely W‐D, W‐S and W‐SH. They refer to the wool fabrics dyed with only the natural dye into the liquor bath (W‐D), to the wool fabric dyed with the addition of 50 g L^−1^ of sodium sulfate (W‐S) and to the wool fabric dyed with the addition of 2% glacial acetic acid and 50 g L^−1^ of sodium sulfate (W‐SH).

### 2.5 Fabrics Characterization and Dyeing Performance Evaluation

The wool‐treated fabric surface morphology was examined with an EVO series (ZEISS) scanning electron microscope (SEM) with an acceleration voltage of 20 kV. The samples were mounted on aluminum specimen stubs with double‐sided adhesive tape and sputter‐coated with gold in rarefied Argon using a Quorum SC 7620 Sputter Coater.

Thermogravimetric analyses (TGA) were carried out using a Mettler Toledo TGA–DSC 1 (Schwerzenbach, Switzerland), in which the fabric samples were inserted within an alumina pan and heated from 30 to 800 °C at a rate of 10 °C min^−1^ under a nitrogen flow of 70 mL min^−1^. The first derivatives of the thermogravimetric profiles (DTG) were constructed to identify the maximum mass‐loss rate temperatures.

Attenuated total reflectance Fourier‐transform infrared (ATR‐FTIR) spectra were recorded to study the vibrational features of the samples and, consequently, chemical bonds. For this purpose, a Thermo Nicolet iZ10 spectrometer (Milan, Italy) equipped with a Smart Endurance TM (ZnSe crystal) was used in the wavenumber range 4000–650 cm^−1^, setting up 32 scans and 4 cm^−1^ band resolution.

The mechanical properties of the samples were determined in accordance with ISO 13934‐1:2013 using an electronic universal testing machine, Dynamometer ZwickRoell ProLine 5 KN (Z005). The samples were tested at a speed of 100 mm min^−1^, with a working distance of 20 mm and a pretension of 2 N. The experiments were conducted in controlled laboratory conditions at 20 °C and 65% relative humidity. The breaking strength and elongation at break were measured in both weft and warp directions, and the mean values were reported.

The dyed samples were analyzed with a Datacolor SF 600 X Spectraflesh with CIE standard illuminant D65, 10°. The reflectance values, R, of the dyed samples were measured and the corresponding color strength, K/S at 520 nm wavelength was calculated with the Kubelka‐Munk correlation, as reported in Equation (1).

(1) $$\frac{K}{S}=\frac{{\left(1-R\right)}^{2}}{2R}$$

where *K* is the absorption coefficient and *S* is the scattering coefficient.

Then, ΔE CIELab color difference values were calculated according to Equation (2).

(2) $$\Delta E=\sqrt{{\left(L-L_{0}\right)}^{2}+{\left(a-a_{0}\right)}^{2}+{\left(b-b_{0}\right)}^{2}}$$

where *L*_0_; *a*_0_; *b*_0_ are the colorimetric parameters of the reference sample according to CIElab color space. L, *a* and *b* are those of the dyed sample. In detail, “*a*” represents the value from green (negative) to red (positive) and “*b*” is the value from blue (negative values) to yellow (positive values), whereas “*L*” is the lightness.^[^ ^68^ ^]^ The reference sample for ΔE values was the plain wool fabric.

Dye exhaustion was evaluated via the calibration line reported in Equation (3). The calibration line was prepared by diluting the mother solution, which had a concentration of 0.5 g L^−1^.

(3) $$A=2.2897c\;\;\;R^{2}=0.9958$$

The washing fastness of the dyed fabrics was evaluated according to the conditions reported in the standard ISO 105‐C06. Detergent ECE water solution (ECE 98 Type A without phosphate supplied by Ausiliari Tessili Srl, Cornaredo, Italy) was prepared at 4 g L^−1^ concentration at 40 °C, stirring for 30 min.

## 3 Results and Discussion

### 3.1 Dyeing Performance

**Table** **1** reports the performances of the different dyeing procedures carried out in this work. In the first column of results, the dye exhaustion data are shown. Dye exhaustion is a crucial parameter because the excess dye not adsorbed by the fibers is discharged and lost into the wastewater. The data reported demonstrate an overall good affinity between the dye and the wool fibers. The dye exhaustion associated with sample W‐D, dyed with only the *Carmine* molecules in the liquor, accounts for 26% dye exhaustion. An increase in dye exhaustion is observed by adding acetic acid and sodium sulfate to favor the dye molecule migration on the fibers. On the other hand, the use of WWHs as mordants brings a significant rise. The application of acid dyes to protein fibers results in a strong ionic linkage between the amino groups of wool that are protonated in an acidic environment and the anionic group of the dye molecules. In addition, Van der Waals forces and hydrogen bonds are established between the –OH groups of the dye molecules and the fibers.^[^ ^69^ ^]^ The higher dye exhaustion of the fabrics associated with the WWHs might be due to the addition of new amino groups that are able to link more dye molecules. As stated by Abo El‐Ola,^[^ ^42^ ^]^ the alkaline hydrolysis of wool breaks the peptide bonds, leading to the formation of low molecular weight proteins and peptides as well as free amino acids in the final product. The differences observed among the samples dyed with the WWHs are not significant. Sample W‐1 evidenced a slightly higher increase in dye exhaustion, which may be attributable to the effect of heat on the WWH fixation on the fibers.

**Table 1:** Dyeing performances of the wool fabrics.

Sample	Dye exhaustion [%]	K/S	Dyed fabric	Washing fastness
W‐D	26.4 ± 1.5	1.0	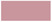	3/4
W‐S	31.2 ± 1.7	1.1	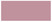	3/4
W‐SH	75.3 ± 1.6	3.0	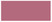	4
W‐1	96.4 ± 0.6	5.6	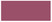	4/5
W‐2	91.4 ± 1.9	4.3	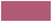	4/5
W‐3	90.3 ± 0.9	3.4	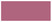	4/5

The washing fastness data reported in Table 1 were determined using the grey scale according to ISO 105‐A03. The range covers values from 1 to 5, where value 1 indicates no fastness to washing, while value 5 is associated with optimum fastness. Values 4/5 were registered for the fabrics dyed with the WWH, confirming that WWHs conferred stability and durability to the fabric dyeing, whereas no significant differences were observed in relation to the different procedures carried out for WWH fixation. The color strength K/S further corroborates that the wool fabrics dyed with the WWHs displayed a higher dye fixation degree on the fibers. In **Table** **2**, the color coordinates of the dyed fabrics are reported, as well as the ΔE CIELab color difference with respect to the plain wool fabric.

**Table 2:** Color coordinates of the dyed samples.

Sample	L	A	b	ΔE
Reference^*)^	85.38	−1.23	8.05	–
W‐D	65.53	21.56	3.60	30.6
W‐S	63.58	20.52	0.30	31.81
W‐SH	53.46	32.76	2.13	47.0
W‐1	44.57	34.95	0.11	55.18
W‐2	49.84	38.33	2.00	53.59
W‐3	52.05	34.23	0.26	49.42

^a)^ plain wool fabric

Regarding *L* values, in the first column, it appears that a general gradual decrease occurs for the dyed fabrics due to the partial absorption of the light by the dye molecules. The plain wool fabric owns the typical color of natural wool; the negative value of coordinate *a* evidences that its color slightly moves toward green, while the positive *b* value is a sign of a yellowish color. The dyed fabrics appear to be darker than the plain, undyed one. *L* values of the fabrics dyed without the WWHs become lower by adding the salt and the acid in the dye bath, meaning that the dyeing process is promoted by an acidic pH. On the other hand, the *L* value of the fabrics dyed with the WWHs is further reduced. The values for coordinate *a* prove that a higher amount of dye is adsorbed by the fibers dyed with the WWHs; *a* shift in the red interval by increasing from ≈33 to ≈38 for sample W‐2. Column *b* does not display a particular trend, since the pigment of *Carmine* dye is red, influencing *a* coordinate more significantly. Coordinates *b* evidence a general decrease in the direction of the blue interval if compared to the reference material. In the last column, the ΔE CIELab data confirm what has been stated previously: overall, they rise when the fabrics are dyed with the WWHs. Among samples W‐1, W‐2 and W‐3, better dyeing performances were observed for samples W‐1 and W‐2, while sample W‐3 had a less performing behavior. This outcome might be due to the positive effect of both temperature‐based treatment prior to the dyeing process in W‐1 and W‐2, whereas in sample W‐3, the WWHs were dispersed into the dyeing bath with the fabric and the dye molecules. In this latter case, a sort of competitive reaction may occur between WWH's reaction with wool or with the dye molecules.

**Figure 2:**
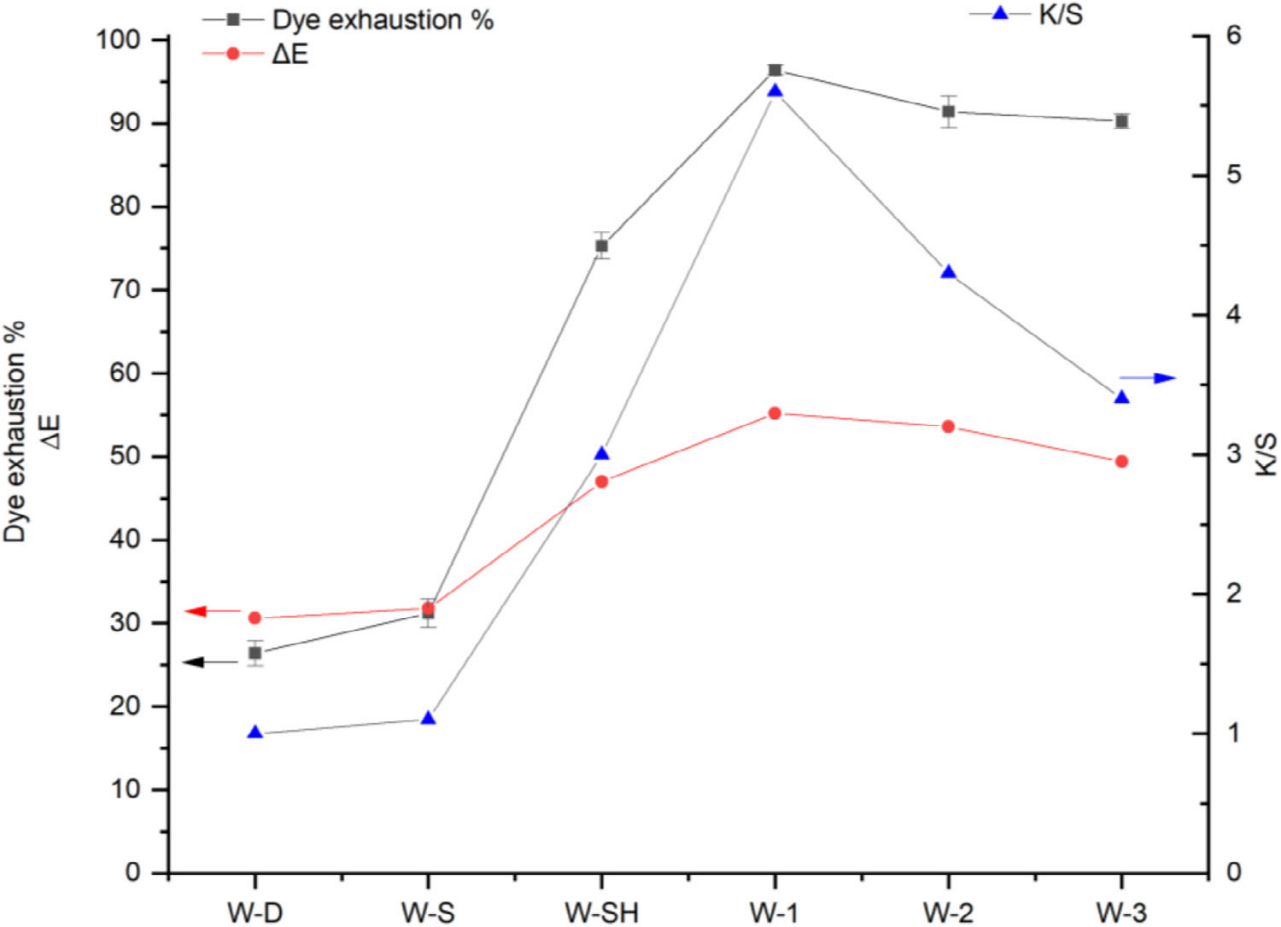
Color performances of the samples dyed under different conditions, where dye exhaustion (%) and ΔE value (adimensional number) use the same scale in the left y axis, while K/S values refer to the right y axis.

In **Figure** **2**, a summarizing graph comparing the color performances of the dyed samples is reported.

### 3.2 Characterization of Treated and Dyed Wool

The characterization of the hydrolysate powder has already been performed in.^[^ ^40^ ^]^ Briefly, the vibrational features evaluated through infrared spectroscopy highlighted the presence of the keratin‐related Amide I and Amide II peaks and the Na_2_SO_4_ signals. The formation of precipitated Na_2_SO_4_ was forecasted and, indeed, the choice of using sulfuric acid to bring WWHs at an acid pH was aforethought since such salt has already been used as a textile dyeing auxiliary.^[^ ^70^, ^71^ ^]^ Moreover, on the previous occasion, DSC analyses showed similarities between the bare keratin and hydrolysate profiles, except for a temperature shift of the peaks related to thermal‐induced denaturation and degradation of the proteins. When the hydrolysate powders were used on cotton, the infrared spectra of such treated fabrics revealed the presence of the impregnation, whereas the DSC profiles were not affected by WWH presence.

**Figure 3:**
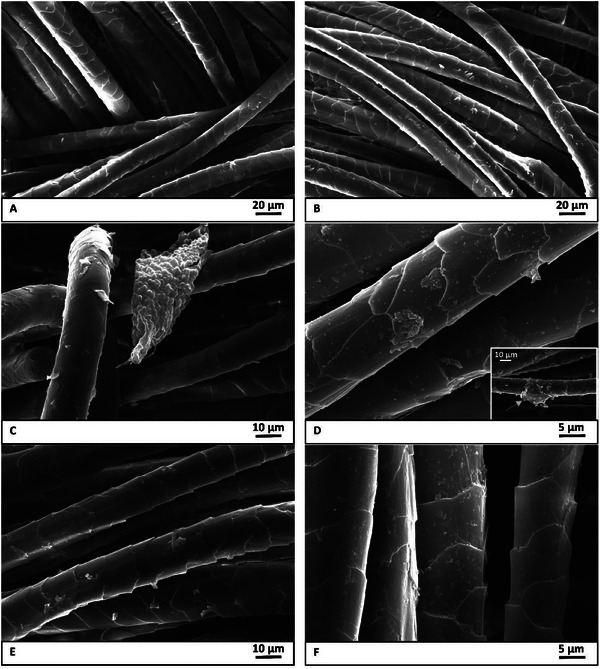
SEM micrographs of A) W‐D, B) W‐S, C) W‐SH, D) W‐1 (with the inset of additional analyzed area), E) W‐2 and F) W‐3.

Herein, we report the physical‐chemical investigations on the various treated wool fabrics prepared in this work. First, the morphology was observed: in **Figure** **3**, a selection of SEM images of dyed wool is displayed.

W‐D and W‐S (Figure 3A,B) showed the typical fiber morphology of wool, characterized by thick cuticle scale edges, higher scale frequency and, thus, higher surface roughness.^[^ ^72^ ^]^ The presence of the dye is not detectable per se. In the case of W‐SH (Figure 3C), more damage to the fibers was observed, probably favored by the addition of free acids in combination with high temperature.^[^ ^73^, ^74^ ^]^ Temperature and pH conditions, indeed, are well‐known tunable and influencing parameters in fabric dyeing processes, also at the industrial scale.^[^ ^74^, ^75^, ^76^, ^77^ ^]^ Regarding the samples treated with WWHs, W‐1 and W‐3 (Figure 3D,F), in contrast to W‐2 (Figure 3E), had some particulate matter on their surface, which can be attributed to the hydrolysate particles. However, W‐1 demonstrated a homogeneous deposition that was, instead, less evenly distributed in the case of W‐3. The factor that could have promoted the interaction of wool with the hydrolysates could be the curing at 180 °C for W‐1, while for W‐3, the simultaneous treatment likely favored the intimate contact between wool and the precipitated hydrolysate. In the case of W‐2, the treatment with WWHs at 100 °C could have caused the swelling of fibers, entrapping more hydrolysate among the fibers and in a lower amount on the surface. Furthermore, some local fiber damages have been visualized, as well (see an illustrative image in the inset of Figure 3D).

**Figure 4:**
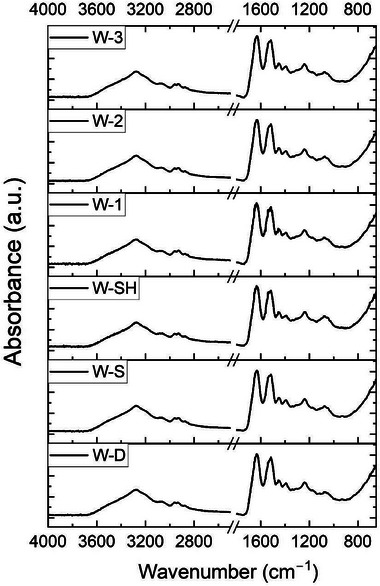
ATR‐FTIR spectra of the samples examined in this work.

The infrared spectra of the samples under investigation resemble that of pure wool and consist of macro‐areas that can be summarized as follows^[^ ^78^ ^]^ (**Figure** **4**):

- 3600–3000 cm^−1^: O‐H and N‐H stretching vibrations (amide‐A and amide‐B bands);
- 2920 and 2850 cm^−1^ peaks: asymmetrical and symmetrical stretching of the CH_2_ and CH_3_ groups;
- 1630 cm^−1^ and at 1525 cm^−1^: C═O stretching vibration and the coupling of the N‐H bending with C═N stretching (Amide I and Amide II bands);
- Band centered at 1235 cm^−1^: the in‐phase combination of C‐N stretching and N‐H bending, with some contribution from C‐C stretching and C═O bending vibrations (Amide III);
- 1200–1000 cm^−1^ (fingerprint region): S‐O stretching vibration band.

All the dyed fabrics showed very similar IR‐vibrational profiles, which is reasonable since both wool and the related hydrolysate are based on keratin.^[^ ^79^ ^]^ However, different features were visible in the Amide I and II signals, especially for W‐SH and W‐1 and can, therefore, be attributed to a major action of acid (from SEM, W‐1 seemed the sample with the greatest impregnation degree after the treatment with the hydrolysate acid dispersion). Indeed, the peak edges are jagged, probably as an index of some modified bonds and interactions, i.e., the formation of NH_3_^+^ and variation in inter‐ and intra‐molecular linkages.^[^ ^80^ ^]^

**Figure 5:**
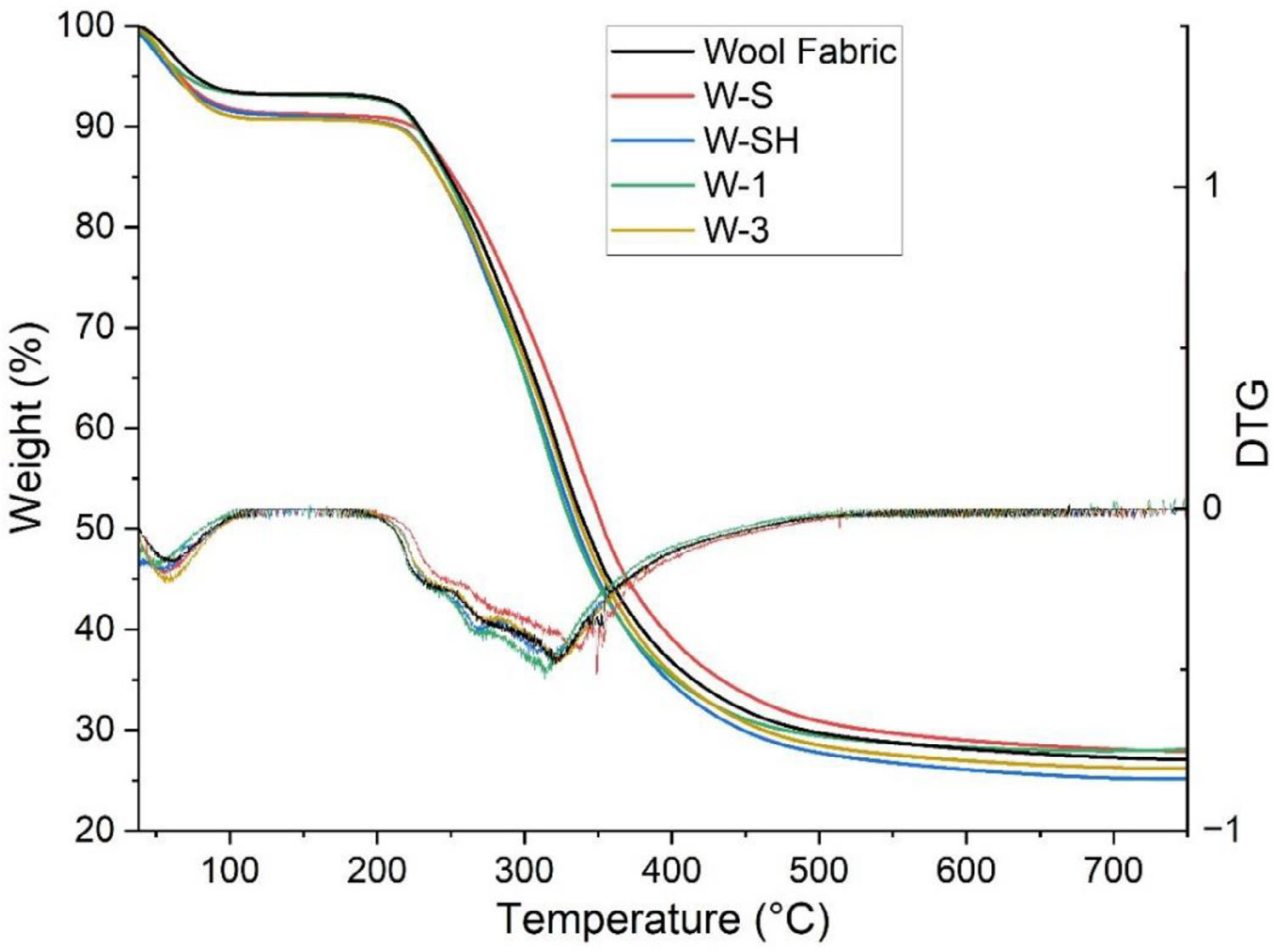
TGA outcomes of selected samples. “Wool Fabric” represents the pristine wool as a reference specimen.

The thermal behavior of the wool samples was evaluated by TGA (in **Figure** **5**, some representative curves are reported). The first step of weight loss is attributed to moisture (<10 wt.%); then, after 210 °C, the thermal degradation takes place and the DTG curves indicate that the main phenomena are similar for all the fabrics analyzed. In particular, the process is multimodal until ≈400 °C. The thermally induced events are related to the disruption of wool hierarchical structure, the decomposition of cystine and terminal amino groups and the decarboxylation.^[^ ^81^ ^]^ The final residues are between 25 and 28 wt.%.

By taking the onset temperatures (the point of intersection of the starting‐mass baseline and the tangent to the TGA curve at the point of maximum gradient^[^ ^82^ ^]^), it has been revealed that the samples dyed with the addition of salt and salt+acidic conditions (W‐S and W‐SH) have started the thermal degradation at more elevated temperatures (6–8 °C higher than pristine wool fabric). Therefore, TGA results seem more influenced by the presence of inorganic substances as “stabilizers”. No other trends could be observed.

**Figure 6:**
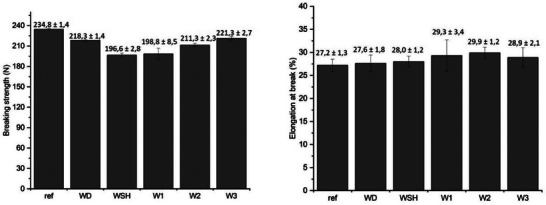
Mechanical properties of the samples: breaking strength (on the left) and elongation at break (on the right). The sample labeled “ref” indicates the reference wool fabric in its pristine form.

Tensile studies were carried out to understand if the mechanical properties of the wool fabrics were affected by the different process conditions (**Figure** **6**). For this reason, the reference sample is the plain, undyed wool fabric.

The tensile strength outcomes reported for the dyed fabrics show a general decrease compared to the undyed fabric. This behavior is due to the dyeing process carried out in a water bath at 100 °C, hypothesizing that minor damage may have occurred to the fibers. The breaking strength of the undyed fabric is 234.8 N while that of W‐D is 218.3 N. Samples W‐SH and W‐1 are less resistant; their breaking registered strengths are, respectively, 196.6 N and 198.5 N. For sample W‐SH, both the temperature of the water‐based dyeing bath and the acidic conditions might have contributed to the decrement in its mechanical properties.^[^ ^83^ ^]^ In sample W‐1, the treatment at 180 °C to promote the fixation of the WWHs might be responsible for the lower breaking strength. The breaking strength of sample W‐3 is very close to that of sample W‐D, namely 221.3 N and 218.3N. Sample W‐3 was indeed dyed in conditions similar to W‐D, with the WWHs dispersed directly into the dyeing bath. Sample W‐2 evidenced a breaking strength of 211.3 N, slightly lower than that of samples W‐D and W‐3. In sample W‐2, the WWHs were applied as a dyeing pre‐treatment at 100 °C for 1 h; therefore, the WWHs did not substantially alter the mechanical properties of the reference material, even though the fabric displayed a slight decrease in mechanical strength.

As a general consideration about elongation at break, the data obtained did not indicate a remarkably different behavior compared to the reference sample.

## 4 Conclusions, Sustainability Awareness and Future Perspectives

Waste wool hydrolysates (WWHs) are a by‐product collected from the alkaline hydrolysis of wasted wool, representing an additional waste. The present work showed the possibility of employing these leftover products as coadjutants in the dyeing of plain wool fabrics. In order to perform an eco‐friendly process, *Carmine* dye was selected as a natural dye. The WWHs were applied to the fabrics as pre‐treatment, prior to the dyeing, and directly into the liquor with the dye. Color performances were evaluated to identify the best procedure in terms of dye exhaustion and washing fastness. For comparison, some wool fabrics were dyed with only the dye in the liquor and by adding salt and acetic acid to favor the dye adsorption on the wool fibers.

The best performances corresponded with the sample pretreated with WWHs and cured at 180 °C (W‐1). Very good color features have been obtained for W‐2, as well, implying a thermal pre‐treatment in the presence of WWHs at 100 °C. It seems that the thermal steps have enhanced the adhesion between the fabric and the WWHs, bringing better dyeing. Regarding this aspect, in a recent LCA study, it has been estimated that the contribution of energy consumption to the overall impact of woolen undershirt production can account for values ranging from 0.2 to 66.7%.^[^ ^84^ ^]^ The energy comprises both heating and electricity, and its footprint can be mitigated using renewable sources.^[^ ^84^ ^]^ Therefore, it is possible to infer that the overall sustainability of the fabrics dyed in the present work should be evaluated by also considering the influence of the heating steps. However, among the samples investigated, W‐3, which was prepared without pre‐treatments, also reached good color features in terms of dye exhaustion, K/S values and washing fastness. Considering the power of heating instruments used (the shaking water bath, the AHIBA equipment and the thermostatic oven for annealing) and the operating time (including the heating ramp), the simultaneous dyeing step planned for W‐3 can save up to 25% of energy. For this reason, it represents an acceptable compromise between performance, time consumption, energy demand and, thus, environmental impact.

Morphology, functional groups and thermal properties of the dyed materials confirmed the efficient attachment of the hydrolysate onto the fabric surface. The mechanical properties of the fabrics were evaluated as well, demonstrating that the procedures employed are not highly detrimental. Only W‐1 (dyed with the WWHs applied as pre‐treatment at room temperature and fixation at 180 °C) and W‐SH (dyed with salt and acid without the WWHs) evidenced a decrease in the breaking strength of the samples if compared to W‐D (dyed without other co‐reactants). This result leads to the hypothesis of a slight effect of free strong acid in the bath and high‐temperature treatment.

**Figure 7:**
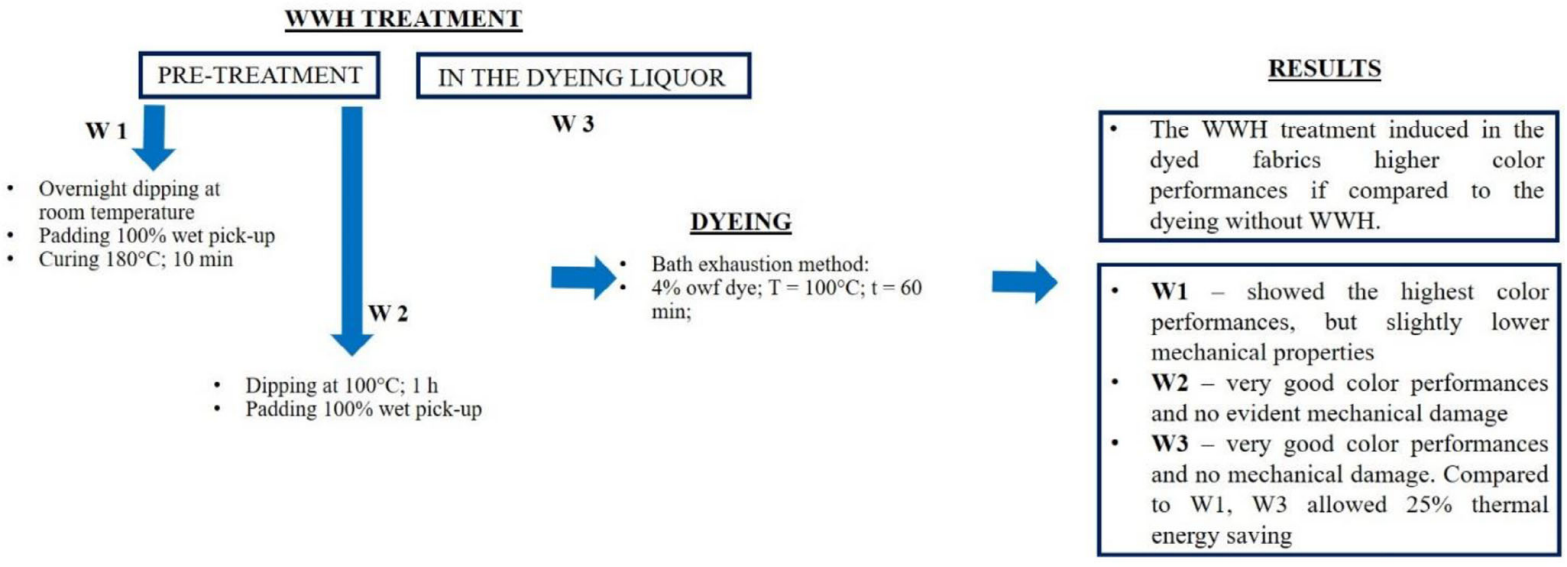
Resume of the dyeing procedures and the main outcomes.

For the sake of clarity, in **Figure** **7**, a schematic representation of the process and the most relevant results obtained are reported.

As a perspective, the introduction of positive charges onto hydrolysate with more eco‐compatible acids, such as acetic and citric ones, will be designed for future studies. Moreover, other kinds of fabric substrates and natural dyes can be adopted to widen the applicability of the presented dyeing methodology.

## Conflict of Interest

The authors declare no conflict of interest.

## Data Availability

The data that support the findings of this study are available from the corresponding author upon reasonable request.
